# FOLFOX as second-line chemotherapy in patients with pretreated metastatic pancreatic cancer from the FIRGEM study

**DOI:** 10.1186/1471-2407-14-441

**Published:** 2014-06-14

**Authors:** Aziz Zaanan, Isabelle Trouilloud, Theofano Markoutsaki, Mélanie Gauthier, Anne-Claire Dupont-Gossart, Thierry Lecomte, Thomas Aparicio, Pascal Artru, Anne Thirot-Bidault, Fanny Joubert, Daniella Fanica, Julien Taieb

**Affiliations:** 1 Department of Gastroenterology and Digestive Oncology, Georges Pompidou European Hospital, AP-HP, 20 rue Leblanc, 75015 Paris, France; 2 University of Paris Descartes, Paris, France; 3 Biostatistic and Quality of Life Unit, Georges François Leclerc Center, Dijon, France; 4 Department of Hepatogastroenterology, Jean Minjoz Hospital, Besançon, France; 5 Department of Hepatogastroenterology, Trousseau Hospital, University of François-Rabelais, Tours, France; 6 Department of Gastroenterology and Digestive Oncology, Avicenne Hospital, HUPSSD, AP-HP, University of Paris 13, Sorbonne Paris Cité, Bobigny, France; 7 Department of Gastroenterology and Digestive Oncology, Jean Mermoz Hospital, Lyon, France; 8 Department of Hepatogastroenterology, Bicêtre Hospital, AP-HP, Kremlin Bicêtre, France

**Keywords:** Pancreatic cancer, FOLFOX, Second-line chemotherapy

## Abstract

**Background:**

FOLFOX second-line treatment seems to be a validated option for patients with pancreatic cancer (PC) progressing after gemcitabine chemotherapy. However, other therapeutics strategy has developed in first-line therapy, as the FIRGEM phase II study that evaluated gemcitabine alone versus FOLFIRI.3 alternating with gemcitabine every two months. The present study assessed the efficacy and safety of FOLFOX after failure of the first-line therapy used in the FIRGEM study.

**Methods:**

In this prospective observational cohort study, we analysed all consecutive patients who received second-line chemotherapy with FOLFOX among 98 patients with metastatic PC included in the FIRGEM study. Progression-free survival (PFS) and overall survival (OS) were estimated from the start of second-line chemotherapy using the Kaplan-Meier method.

**Results:**

Among 46 patients who received second-line chemotherapy, 27 patients (male, 55%; median age, 61 years; performance status (PS) 0–1, 44%) were treated with FOLFOX after progression to first-line gemcitabine alone (n = 20) or FOLFIRI.3 alternating with gemcitabine (n = 7). Grade 3 toxicity was observed in 33% of patients (no grade 4 toxicity). At the end of follow-up, all patients had progressed and 25 had died. No objective response was observed, and disease control rate was 36%. Median PFS and OS were 1.7 and 4.3 months, respectively. In multivariate analysis, PS was the only independent prognostic factor. For patients PS 0–1 versus 2–3, median PFS was 3.0 versus 1.2 months (log rank, p = 0.002), and median OS was 5.9 versus 2.6 months (log rank, p = 0.001).

**Conclusions:**

This study suggests that FOLFOX second-line therapy offered interesting efficacy results with an acceptable toxicity profile in metastatic PC patients with a good PS.

## Background

Pancreatic cancer (PC) accounts for approximately 2-3% of all malignant neoplasms worldwide, but is the fifth cause of cancer-related death in Western countries [1]. At the time of diagnosis, about half of patients have metastatic disease, with a very poor prognosis and a median overall survival (OS) of about 6 months [2]. Palliative chemotherapy is most often the only treatment option for this group of patients. Until recently, gemcitabine was considered as the standard therapy based on a randomised phase III trial showing a better clinical benefit and survival compared to 5-fluorouracil (5FU) chemotherapy [3]. Since then, several randomised studies have been performed to improve the efficacy of first-line chemotherapy by combining gemcitabine with other cytotoxic or molecular targeted agents [4]. No significant improvement in OS has been observed with any gemcitabine doublet, except for erlotinib, which provided a relatively low benefit [5]. More recently, two randomised phase III trials comparing gemcitabine with FOLFIRINOX and gemcitabine with gemcitabine plus nab-paclitaxel in first-line chemotherapy have shown a significant improvement in objective response rate (ORR), progression-free survival (PFS) and OS in patients with metastatic PC [6,7]. However, these treatments are indicated only for selected patients with good performance status (PS) because of a higher rate of severe toxicities than with gemcitabine alone.

After first-line chemotherapy failure, there is no standard second-line therapy. At this time, only one randomised phase III trial including forty-six patients who had become resistant to gemcitabine suggested that the combination of 5FU and oxaliplatin as second-line therapy is superior to best supportive care (BSC) [8].

Over several years, our team developed the FOLFIRI.3 regimen in first-line treatment of metastatic PC, with interesting results in a phase II study [9]. Subsequently, we performed a randomised phase II trial, named FIRGEM, to evaluate gemcitabine alone versus FOLFIRI.3 alternating with gemcitabine every two months in patients with previously untreated metastatic PC [10]. After 23 months of median follow-up, patients receiving sequential treatment had a higher PFS rate at 6 months (31% versus 49%) and 1 year (11% versus 23%), a higher ORR (11% versus 40%) and manageable toxicities [10]. Based on these results, this sequential treatment strategy should be compared with FOLFIRINOX in a phase III trial.

In the present prospective “Association des gastroentérologues oncologues” (AGEO) study, we report the efficacy and tolerability of second-line FOLFOX chemotherapy in patients included in the FIRGEM trial with PC that progressed after first-line gemcitabine or sequential treatment.

## Methods

### Patients

This study was reviewed and approved by the Pitié-Salpêtrière Hospital Ethics Committee for all participing centres. In this prospective observational cohort study, we analysed all consecutive patients who received second-line chemotherapy by FOLFOX among patients with metastatic PC included from October 2007 and March 2011 in the FIRGEM trial [10]. All patients had measurable disease. After disease progression under first-line chemotherapy with gemcitabine alone or FOLFIRI.3 alternating with gemcitabine, the second-line treatment and protocol were left to the investigator’s discretion. All patients were over 18 years of age and provided signed written informed consent. The study was registered with EudraCT (N° 2006-005703-34).

### Treatment

The FOLFOX regimen consists of oxaliplatin 85 mg/m^2^ in 2 hours infusion, folinic acid 400 mg/m^2^ in 2 hours infusion, followed by 5FU bolus 400 mg/m^2^, then by 5FU continuous infusion 2400 mg/m^2^ in 46 hours. The chemotherapy was administered every two weeks until the patients declined further doses or until disease progression or limiting toxicities.

Before each cycle of administration, a physical examination and laboratory tests were performed, in order to evaluate the ECOG PS status of the patient and treatment tolerability.

### Study evaluations

Tumor evaluation was performed every two months after the first day of treatment (or earlier in patients with suspected disease progression) and consisted of a physical examination, laboratory tests including determination of serum carcinoembryonic antigen (CEA) and carbohydrate antigen 19–9 (CA 19–9), and computed tomography (CT) scans. Tumor response was assessed according to the Response Evaluation Criteria in Solid Tumors (RECIST) criteria [11]. Objective response rate was defined as the percentage of patients who had a complete or partial response. Toxicity was graded before each cycle of chemotherapy according to the National Cancer Institute Common Terminology Criteria for Adverse Events (version 3.0). For this study, the cutoff date of analysis was March 2012.

### Statistical analysis

The statistical tests were performed with STATA V11 software (STATA Corp, College Station, TX). Progression-free survival was calculated from the date of second-line therapy initiation to the date of progression or death (all causes), whichever came first. Surviving patients without disease progression were censored at the last visit date. Overall survival was calculated from the date of second-line therapy initiation to death (all causes). Surviving patients were censored on the last follow-up date. Progression-free and overall survival curves were estimated by the Kaplan-Meier method and compared with the log-rank test. For univariate and multivariate analyses, Cox’s proportional hazards model was used to calculate hazard ratios (HR) with 95% confidence intervals (CI) for PFS and OS. All tests were two-sided and a p-value < 0.05 was considered to be statistically significant.

## Results

### Patient characteristics

Among the 98 patients included in the FIRGEM study, 46 (47%) were treated with second-line chemotherapy: 27 received FOLFOX and 19 other regimens. Among the 27 patients treated with FOLFOX in second-line chemotherapy, the median age was 61.4 years (range, 47.6-74.1 years) and first-line chemotherapy was FOLFIRI.3 alternating with gemcitabine for 7 patients (26%) and gemcitabine alone for 20 patients (74%). The others characteristics are summarised in Table 1.

**Table 1:** Patients characteristics

**No. of patients**	27
**Median age [range], years**	61.4 [47.6 - 74.1]
**Gender**	
Male	15 (55.6%)
Female	12 (44.4%)
**ECOG performance status score**	
0	5 (18.5%)
1	7 (25.9%)
2	9 (33.3%)
3	3 (11.1%)
Unknown	3 (11.1%)
**First line chemotherapy**	
FOLFIRI.3 alternating with Gemcitabine	7 (25.9%)
Gemcitabine	20 (74.1%)
**Pancreatic tumor location**	
Head	14 (51.8%)
Body	6 (22.2%)
Tail	6 (22.2%)
Multicentric	1 (3.7%)
**Metastatic sites**	
Liver	20 (74.1%)
Peritoneum	8 (29.6%)
Lung	6 (22.2%)
Distant lymph nodes	2 (7.4%)
**No. of metastatic sites involved**	
1	19 (70.4%)
2	7 (25.9%)
≥ 3	1 (3.7%)
**Baseline CA 19–9 level**	
≤ 37 UI/ml*	2 (7.4%)
> 37 UI/ml	19 (70.4%)
Unknown	6 (22.2%)
**No. of cycle**	
Mean	6
Median [range]	4 [1–28]

*Abbrevations:ECOG* Eastern Cooperative Oncology Group;

*upper limit of normal value.

A total of 168 chemotherapy cycles were administered in FOLFOX second-line treatment (median, 4; range, 1–28).

### Toxicity

All patients treated with FOLFOX in second-line chemotherapy were evaluated for toxicity (Table 2). No grade 4 haematological or non-haematological toxicities were observed. Nine patients (33%) developed at least one grade 3 adverse event. The main grade 3 toxicities were asthenia (15%) and thrombocytopenia (11%). Severe sensory neuropathy was observed in 2 (7%) patients. No febrile neutropenia or toxic death was reported.

**Table 2:** Toxicities

	**No. of Patients (%)**			
	**Grade 1**	**Grade 2**	**Grade 3**	**Grade 4**
**Hematologic event**				
Neutropenia	2 (7.4)	3 (11.1)	2 (7.4)	0 (0.0)
Febrile neutropenia	-	-	0 (0.0)	0 (0.0)
Thrombocytopenia	12 (44.4)	1 (3.7)	3 (11.1)	0 (0.0)
Anemia	10 (37.0)	5 (18.5)	2 (7.4)	0 (0.0)
**Nonhematologic event**				
Asthenia	7 (25.9)	11 (40.7)	4 (14.8)	0 (0.0)
Nausea/Vomiting	10 (37.0)	6 (22.2)	0 (0.0)	0 (0.0)
Mucitis	3 (11.1)	0 (0.0)	0 (0.0)	0 (0.0)
Diarrhea	6 (22.2)	4 (14.8)	0 (0.0)	0 (0.0)
Sensory neuropathy	10 (37.0)	2 (7.4)	2 (7.4)	0 (0.0)

Toxicities were evaluated according to National Cancer Institute Common Terminology Criteria for Adverse Events (version 3.0).

### Tumor response and survival

Tumor response was evaluated in 22 of the 27 patients included. Evaluation was not performed in 4 patients because of early death due to rapid clinical progression of the disease after 1 (n = 2) and 2 (n = 2) cycles, and in 1 patient because of limiting toxicity after two cycles of chemotherapy which leaded to treatment discontinuation. No objective response was recorded and 8 patients (36%) had disease stabilisation (Table 3).

**Table 3:** Tumor Response and Survival

	**No. of Patients (%)**
**Tumor response**	
Complete response	0 (0.0)
Partial response	0 (0.0)
Stable disease	8 (36.4)
Progression disease	14 (63.6)
**Median progression free survival (months) [95% CI]**	1.7 [1.0 - 2.5]
**Median overall survival (months) [95% CI]**	4.3 [2.2 - 5.9]

Tumor response was evaluated in 22 patients among the 27 patients included. Evaluation could not be performed for 4 patients because of early death and for 1 patient because of limiting toxicity after two cycles.

**Figure 1:**
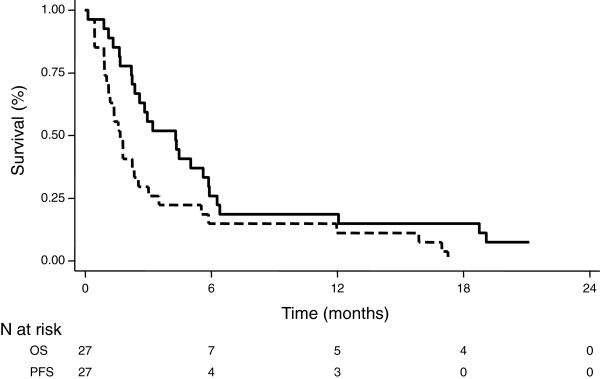
Kaplan-Meier survival analysis of progression-free survival (PFS, dashed dark line) and overall survival (OS, solid dark line) from the start of FOLFOX second-line chemotherapy.

At the end of follow-up, PC had progressed in all patients, and 25 of them died. Median PFS from the start of the second-line chemotherapy was 1.7 months (95% CI, 1.0 - 2.5 months) (Figure 1). The 6-month and 1-year PFS rates were 14.8% (95% CI, 4.7 – 30.5%) and 11.1% (95% CI, 2.8 – 25.9%), respectively.

Median OS from the start of the second-line chemotherapy was 4.3 months (95% CI, 2.2 – 5.9 months) (Figure 1). The 6-month and 1-year OS rates were 25.9% (95% CI, 11.5 – 43.1%) and 18.5% (95% CI, 6.8 – 34.8%), respectively.

Median OS from the start of first-line therapy was 12.6 months (95% CI, 7.1 - 26.3 months) for patients treated with FOLFIRI.3 alternating with gemcitabine, and 8.5 months (95% CI, 6.9 – 10.6 months) for patients treated with gemcitabine alone.

### Prognostic factors

In univariate analysis, we evaluated several factors potentially affecting PFS or OS in FOLFOX second-line chemotherapy (sex, age, ECOG PS, first-line chemotherapy protocol, PFS in first-line chemotherapy, number of metastatic sites and baseline CA 19–9 level). For PFS, we observed that ECOG PS [0–1 versus 2–3; HR = 4.00 (95% CI, 1.5 – 10.4), p < 0.01] and PFS in first-line chemotherapy [>6 versus ≤ 6 months; HR = 2.85 (95% CI, 1.0 - 7.9), p = 0.045] were significantly associated with longer PFS in second-line FOLFOX chemotherapy. For OS, we observed that only ECOG PS [0–1 versus 2–3; HR = 4.58 (95% CI, 1.7 – 12.1), p < 0.01] was significantly associated with longer OS in second-line FOLFOX chemotherapy. The first-line chemotherapy protocol used (gemcitabine alone versus FOLFIRI.3 alternating with gemcitabine) was not correlated with PFS or OS in second-line FOLFOX chemotherapy.

**Figure 2:**
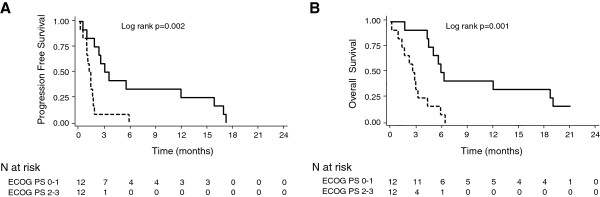
Kaplan-Meier survival analysis of progression-free survival (A) and overall survival (B) from the start of FOLFOX second-line chemotherapy according to ECOG PS status (0–1, solid dark line) (2–3, dashed dark line).

In multivariate analysis, ECOG PS (0–1 versus 2–3) was an independent prognostic factor associated with PFS in second-line FOLFOX chemotherapy [HR = 2.88 (95% CI, 1.0 – 7.7), p = 0.03], while PFS in first-line chemotherapy (>6 versus ≤ 6 months) was not statistically significant [HR = 3.04 (95% CI, 0.8 – 11.0), p = 0.09]. For OS, ECOG PS (0–1 versus 2–3) remains an independent prognostic factor [HR = 3.37 (95% CI, 1.2 – 9.1), p = 0.02]. For ECOG PS 0–1 versus 2–3 patients, the median PFS was 3.0 (95% CI, 0.9 – 15.9) versus 1.2 (95% CI, 0.5 – 1.7), and the median OS was 5.9 (95% CI, 4.3 – 19.1) versus 2.6 (95% CI, 0.9 – 4.3) (Figure 2).

## Discussion

PC has been considered as chemo-refractory disease for a long time. Patients with metastatic PC that progresses after 5FU-based chemotherapy have had a little opportunity to receive second-line chemotherapy, mainly because of poor PS prohibiting further therapy. Gemcitabine first-line chemotherapy has been a major advance in the medical management of metastatic PC, allowing improvement of cancer-related symptoms, clinical benefit and a modest survival advantage. A significant percentage of patients whose PC progresses on gemcitabine chemotherapy still have a relatively good performance status and could benefit from a second-line therapy. Several studies including a relatively small number of patients have evaluated different anti-tumor drugs in this setting, mainly cisplatin [12], irinotecan [13,14], taxanes [15] and raltitrexed [16]. None of these drugs has yielded a significant survival benefit after gemcitabine-based first-line chemotherapy [4].

Oxaliplatin is one of the main drugs used in gastrointestinal cancer treatment. For metastatic PC, a phase II randomised study suggested that a combination of oxaliplatin with 5FU was associated with better ORR and longer survival than 5FU alone or oxaliplatin alone in first-line treatment [17]. After gemcitabine-based resistance, the 5FU/oxaliplatin combination as second-line therapy has been evaluated in seven prospective phase II studies (Table 4). These studies, which included between 18 and 41 patients, showed an ORR of 0% to 7% [14,18-22], except for Tsavaris et al. who reported an ORR of 23% [23]. The median OS in these studies ranged from 1.3 to 6 months approximately (Table 4). Comparison of ORR and survival results from these studies should be interpreted with caution because of the various doses or schedules of 5FU/oxaliplatin used, and because of heterogeneous populations, including various PS or disease stages (locally advanced or metastatic disease) (Table 4).

**Table 4:** Oxaliplatin and fluoropyrimidine as second-line chemotherapy in advanced pancreatic cancer

**Authors**	**Prospectives Studies**	**No. of Patients**	**First-line therapy**	**Regimen**	**ECOG Performance status (PS)**	**ORR**	**Median TTP**	**Median** **OS**	**Main Toxicities grade 3-4**
Androulakis N et al., 2005 [18]	Phase II	18	Gemcitabine-based, n=18	oxali 180 mg/m2 q3weeks	PS 0, n=4 PS 1, n=9 PS 2, n=5	0%	_	3.5 mo	Diarrhoea 5% Vomiting 5% (Neutropenia 0%)
Tsavaris N et al., 2005 [23]	Phase II	30	Gemcitabine, n=30	oxali 50 mg/m2 D1,Leucovorin 50 mg/m2 D1,5FU 500 mg/m2 D1,q1 week	KPS 100-80%, n=10 KPS 70-50%, n=20	23%	22 wks	25 wks	Leucopenia 16% Diarrhoea 14%
Mitry E et al., 2005 [19]	phase II	18	Oxaliplatin, n=10 5FU, n=8	oxaliplatin 130 mg/m2 D1,5FU 1000 mg/m2 D1-D4,q3weeks	PS 0-1, n=4PS 2, n=7 PS > 2, n=4 Unknown, n=3	0%	0.9 m	1.3 mo	Neutropenia 19% Anemia 25% Asthenia 56%
Xiong HQ et al., 2008 [20]	Phase II	41	_	oxaliplatin 110–130 mg/m2 D1,capecitabine 1.5-2 g/m2 D1-D14q3week	PS 0, n=4 PS 1, n=16 PS 2, n=8	3%	9.9 wks*	23 wks	Asthenia 13% Diarrhoea 5%
Pelzer U et al., 2009 [21]	Phase II	37	Gemcitabine, n=37	oxaliplatin 85 mg/m2 D8, 22 folinic acid 500 mg/m2 D1,8,15,225FU 2600 mg/m2 D1,8,15,22 q6weeks	KPS 90-60%, n=37	6%	12 wks	22 wks	Nausea/vomiting 11% Diarrhoea 12%
Novarino A et al., 2009 [22]	Phase II	23	Gem alone, n=13 Gem/5FU/cisplatin, n=5 Gem/5FU, n=4 Gem/oxaliplatin, n=1	oxaliplatin 40 mg/m2 D1,8,15 leucovorin 250 mg/m2 D1,8,15 5FU 500 mg/m2 D1,D8,15 q4weeks	PS 0, n=6 PS 1, n=11 PS 2, n=6	0%	11.6 wks	17.1 wks	Diarrhoea 9% (Neutropenia 0%)
Yoo C et al., 2009 [14]	Randomised phase II (versus FOLFIRI3)	30	Gem alone, n=2Gem/cap, n=26 Gem/erlotinib, n=2	oxaliplatin 85 mg/m2 D1 leucovorin 400 mg/m2 D1 5FU 2000 mg/m2 D1,D2 q2weeks	PS 0, n=5 PS 1, n=24 PS 2, n=1	7%	6 wks*	14.9 wks	Neutropenia 20% Asthenia 14%
Pelzer U et al., 2011 [8]	Randomised phase III (versus BSC)	23	Gemcitabine, n=23	oxaliplatin 85 mg/m2 D8, 22 folinic acid 200 mg/m2 D1,8,15,22 5FU 2000 mg/m2 D1,8,15,22 q6weeks	KPS 100-90%, n=12 KPS 80-70%, n=11	_	_	4.82 mo (vs 2.3 mo BSC)	Diarrhoea 9% (Neutropenia 0%)

*Abbreviations:ECOG* Eastern Cooperative Oncology Group, *KPS* Karnofsky Performance status, *BSC* Best Supportive Care, *TTP* Time To Progression, *PFS* Progression Free Survival, *OS* Overall Survival.

*Evaluation according to PFS.

To our knowledge, only one phase III randomised study has compared 5FU with oxaliplatin (OFF regimen) versus BSC after tumor progression on first-line gemcitabine chemotherapy [8]. Among the 46 patients out of 165 planned, those treated with OFF had a significantly longer median OS [4.82 months (95% CI, 4.29 – 5.35 months)] than patients who received BSC [2.30 months (95% CI, 1.76 – 2.83 months)] [HR = 0.45 (95% CI, 0.24 – 0.83), p = 0.008]. The median OS from the start of first-line gemcitabine therapy was 9.09 months (95% CI, 6.97 – 11.21 months) for the OFF group and 7.9 months (95% CI, 4.95 – 10.84 months) for the BSC group [HR = 0.50 (95% CI, 0.27 – 0.95), p = 0.031] [8]. Although stopped prematurely because BSC alone was no longer accepted by participating centres, this phase III study provided for first time evidence of the survival benefit of second-line chemotherapy for patients with advanced PC.

In our study, the survival results of patients receiving FOLFOX as second-line chemotherapy seem to be in line with those published by Pelzer et al. [8]. Toxicity was manageable without grade 4 side effects, and we noted no toxic death. As shown in other studies, we observed that ECOG PS was an independent prognostic factor [23]. The median OS for PS 0–1 patients was 5.9 months compared with 2.6 months for PS 2–3 patients. As suggested by others studies, PFS in first-line therapy seemed to impact the PFS in second-line chemotherapy [16,23,24], though this result was not statistically significant in our multivariate analysis (p = 0.09).

Our study is the first to evaluate the FOLFOX regimen in second-line treatment after FOLFIRI.3 based-chemotherapy. However, these results should be interpreted with caution because of the relatively small number of patients included. Notably, we observed that only approximately 25% (7 of 27) of patients treated with second-line FOLFOX had received FOLFIRI.3 alternating with gemcitabine in first-line chemotherapy (Table 1). In this study, investigators preferentially proposed FOLFOX after gemcitabine failure, while gemcitabine-based treatment was preferred after failure of the sequential arm. Moreover, among patients who received the sequential arm in first-line, 7 were still being treated in first-line (compared with none in gemcitabine arm) at the cutoff date of analysis.

In recent years, two randomised phase III trials have shown that FOLFIRINOX or gemcitabine plus nab-paclitaxel improved survival compared with gemcitabine alone in patients with metastatic PC [6,7]. However, these treatments are indicated only for selected patients because of a higher incidence of grade 3 or 4 toxicities. Moreover, limiting neurotoxicity of oxaliplatin or nab-paclitaxel could require therapy to be changed or stopped in patients who are still responding and have a good PS. The development of a strategy to limit toxicity could be interesting for treatment of metastatic PC patients. In the FIRGEM phase II trial, we evaluated FOLFIRI.3 alternating with gemcitabine in order to prevent the phenomena of cross-drug resistance and limiting toxicity inherent to any treatment [10]. Furthermore, the FIRGEM strategy, which does not lead to neurotoxicity, allows patients to be given second-line treatment with FOLFOX.

## Conclusions

This prospective study suggests that FOLFOX second-line therapy allows an interesting antitumor efficacy with a manageable toxicity profile in metastatic PC patients with a good PS who had been pretreated in the FIRGEM study. FOLFOX is a validated option in second-line therapy, which may be offered to patients who previously received first-line treatment without limiting neurotoxicity. The sequential FIRGEM first-line treatment followed by FOLFOX should be compared with FOLFIRINOX followed by gemcitabine in a phase III randomised study.

## Competing interests

The authors declare that they have no competing interests.

## Authors’ contributions

AZ, IT, MG and JT conceived the study, collected, analyzed and interpreted the data and drafted the manuscript. TM, ACDG, TL, TA, PA, ATB, FJ and DF made substantial contributions to data collection and analysis. All authors approved the final version of the manuscript.

## Pre-publication history

The pre-publication history for this paper can be accessed here:

http://www.biomedcentral.com/1471-2407/14/441/prepub
